# Epithelial damage and ageing: the perfect storm

**DOI:** 10.1136/thorax-2024-222060

**Published:** 2025-05-27

**Authors:** Richard J Hewitt, Laurence Pearmain, Elisavet Lyka, Jennifer Dickens

**Affiliations:** 1 King’s Centre for Lung Health, King’s College London, London, UK; 2 Faculty of Biology, Medicine and Health, University of Manchester, Manchester, UK; 3 ILD Unit, North West Lung Centre, Wythenshawe Hospital, Manchester Foundation Trust, Manchester, UK; 4 Cambridge University Hospitals NHS Foundation Trust, Cambridge, UK; 5 Cambridge Institiute for Medical Research, University of Cambridge, Cambridge, UK; 6 Royal Papworth NHS Foundation Trust, Cambridge, UK

**Keywords:** Idiopathic pulmonary fibrosis, Interstitial Fibrosis, Innate Immunity

## Abstract

**Background:**

Idiopathic pulmonary fibrosis (IPF) is a progressive disease of lung parenchymal scarring that is triggered by repeated microinjury to a vulnerable alveolar epithelium. It is increasingly recognised that cellular ageing, whether physiological or accelerated due to telomere dysfunction, renders the epithelium less able to cope with injury and triggers changes in epithelial behaviour that ultimately lead to the development of disease.

**Aims:**

This review aims to highlight how, with increasing age, the alveolar epithelium becomes vulnerable to exogenous insults. We discuss the downstream consequences of alveolar epithelial dysfunction on epithelial phenotype, alveolar repair and pro-pathogenic interactions with other alveolar niche-resident cell types which drive IPF pathogenesis.

**Narrative:**

We highlight how a wide array of cellular mechanisms that maintain cellular homeostasis become dysfunctional with ageing. Waning replicative capacity, genomic stability, mitochondrial function, proteostasis and metabolic function all contribute to a phenotype of vulnerability to ‘second hits’. We discuss how in IPF the alveolar epithelium becomes dysfunctional, highlighting changes in repair capacity and fundamental cellular phenotype and how interactions between abnormal epithelium and other alveolar niche-resident cell types perpetuate disease.

**Conclusions:**

The ageing epithelium is a vulnerable epithelium which, with the cumulative effects of environmental exposures, fundamentally changes its behaviour towards stalled differentiation, failed repair and profibrotic signalling. Further dissection of aberrant epithelial behaviour, and its impact on other alveolar cell types, will allow identification of novel therapeutic targets aimed at earlier pathogenic events.

## Introduction

 Idiopathic pulmonary fibrosis (IPF) is the most common fibrosing interstitial lung disease (ILD) and remains a devastating, universally fatal condition. It is now understood that IPF is triggered by the aberrant response of vulnerable alveolar epithelial type 2 (AT2) cells exposed to repeated microinjury, largely from airborne insults.^1^ Driven by these abnormal epithelial cells, complex pathogenic cell–cell interactions and signalling within defined lung tissue microenvironments termed ‘niches’ result in activation of fibroblasts and myofibroblasts. This leads to excessive extracellular matrix (ECM) deposition, causing progressive fibrosis, architectural remodelling and impaired gas exchange. Why these AT2 cells become dysfunctional remains incompletely understood, but several intrinsic and extrinsic factors can lead to epithelial vulnerability and reduced ability to respond appropriately to injury. One of the most important factors is the impact of ageing; indeed, age is the most important risk factor for IPF development.^2^ Ageing-related biological signatures are almost all seen as upregulated in IPF, including the presence of short telomeres, a hallmark of ‘old’ cells.^3^ Here, we discuss how ageing impacts on epithelial function, leaving it susceptible to a dysregulated response to injury, and explain the consequences of aberrant AT2 cell behaviour in triggering IPF.

## Evolution of a vulnerable epithelium: ageing and genetic contributors

**Figure 1:**
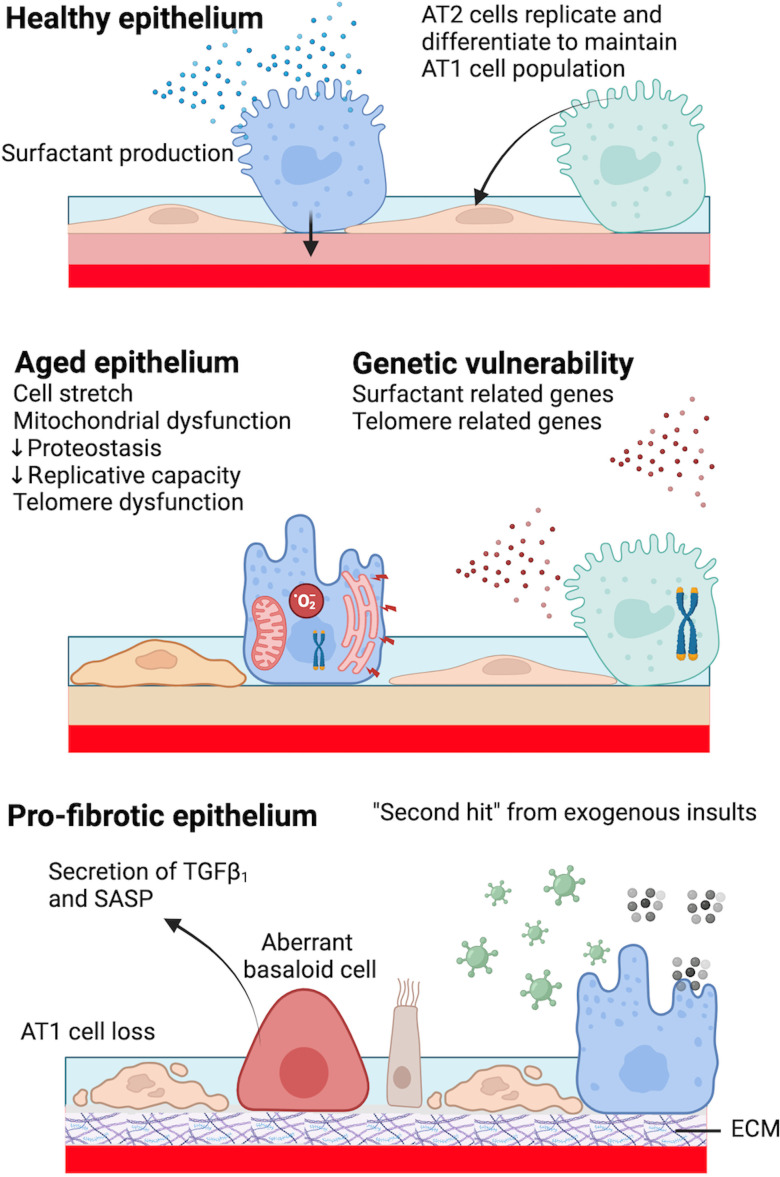
Features of healthy, aged and profibrotic alveolar epithelium. In health, AT2 cells primarily exist to secrete surfactant and act as progenitor cells for alveolar repair following injury (top panel). Epithelium can be rendered vulnerable through the effects of ageing and/or genetic factors (middle panel). A profibrotic epithelium is characterised by loss of AT1 cells, abnormal/stalled differentiation of AT2 cells which secrete profibrotic factors and the appearance of cells with more proximal features (bottom panel). Created in BioRender. AT1, alveolar type 1 epithelial cell; AT2, alveolar type 2 epithelial cell; SASP, senescence-associated secretory phenotype; ECM, extracellular matrix.

In health, the behaviour of alveolar epithelium changes dramatically with advancing age. A complex interplay of genetic, phenotypic, signalling pathway and quality control mechanism changes renders the aged epithelium vulnerable and less well able to handle exogenous insults (figure 1). In this section, we describe ageing-associated changes to ‘intrinsic’ biological processes within AT2 cells that can result in cellular dysfunction, and the resulting ‘extrinsic’ consequences on AT2 cell phenotype and function. We then highlight the role of inherited genetic variants and lung mechanics on AT2 cell dysfunction in the ageing lung.

### Intrinsic cellular dysfunction

#### Replicative capacity

There is a limit to how many times a cell can divide.^4^ This is largely determined by the length of telomeres, the protective caps on the ends of chromosomes which exist to prevent DNA damage during cell division but become shorter with each cell division. Once they become critically shortened, replication ceases and senescence ensues. Thus, with ageing, the proliferative capacity of the global AT2 cell population decreases, with cells variably approaching senescence depending on the demand placed on them for regeneration following alveolar injury.^5^

#### Genomic instability

Cells with very short telomeres may develop telomere or chromosome fusions, creating catastrophic genomic instability.^6^ More modest mutations may be caused by oxidative stress and perpetuated by ailing DNA repair mechanisms. Global changes to DNA methylation and histone modification that occur with ageing may cause epigenetic modification of cell type-specific gene expression, including effects on cell cycle and DNA damage responses.^7 8^

#### Mitochondrial dysfunction

Mitochondrial dysfunction is a prominent feature of ageing AT2 cells. Structural changes are accompanied by a reduction in ATP production and, importantly, an increase in reactive oxygen species (ROS) production as the electron transport chain (ETC) becomes less efficient.^9^ Mitochondrial DNA (mtDNA) is highly sensitive to oxidative damage, causing mutations to accumulate which affects mitochondrial protein production and further exacerbates ETC dysfunction in a positive feedback loop.^10^ There is declining capacity for mitophagy, which is a specialised form of autophagy (a process by which cells selectively remove damaged/dysfunctional cytoplasmic contents). This means that damaged mitochondria fail to be removed and accumulate in AT2 cells, contributing to cellular stress responses and the low-level inflammation seen in ageing known as ‘inflammaging’.^11^

#### Oxidative stress

Oxidative stress occurs when the generation of ROS exceeds the ability of antioxidant enzymes to degrade them, resulting in oxidation and altered function of proteins, lipids and nucleic acids.^12^ ROS production increases with age primarily due to mitochondrial dysfunction,^13^ but also the cumulative effects of environmental exposures (eg, cigarette smoke, particulate matter) and inflammaging. With declining metabolic function, the ability of aged AT2 cells to clear ROS is impaired,^14^ further exacerbating the problem.

#### Proteostasis

Mechanisms designed to maintain proteostasis protect against the accumulation of dysfunctional or misfolded proteins. With increasing age, the expression of endoplasmic reticulum (ER)-resident protein folding chaperones decreases, and chaperone oxidation further impairs their functionality.^15^ The intracellular burden of abnormal proteins increases, but their clearance (via proteasomal or autophagic degradation) also becomes less efficient with age.^16 17^ This leaves AT2 cells vulnerable to entering a state of ‘ER stress’ and triggering an unfolded protein response (UPR) which acts to restore proteostasis via changes to transcription and translation to optimise protein folding capacity. Though a protective measure when transient, prolonged ER stress has detrimental effects on function, phenotype and survival.^18^

#### Metabolic dysregulation and nutrient sensing

AT2 cell metabolism is determined by nutrient, oxygen and energy availability, so close nutrient sensing is crucial to optimal cellular function. Fundamental nutrient sensors and their effector pathways (IGF-1, AMPK, mTOR, sirtuins) become dysregulated with age and have fundamental effects on not just metabolism but autophagy, proteostasis, the cell cycle and apoptosis.^19^

#### Senescence

Consequences of AT2 cellular ageing include telomere shortening, epigenetic dysregulation, oxidative stress, impaired autophagy and mitochondrial dysfunction; together they lead to cellular senescence, illustrated by an excess of senescent AT2 cells in aged lungs.^20^ Senescent cells express cell cycle arrest proteins (eg, p16 and p21) and display fundamentally changed behaviour. They become resistant to apoptosis and develop a senescence-associated secretory profile (SASP),^21^ which influences the alveolar niche in both an autocrine and paracrine fashion.

### Extrinsic cellular dysfunction: functional and phenotypic changes

#### Stem cell exhaustion

AT2 cells function as stem cells in the alveolar niche, effecting alveolar repair following injury by differentiating to replenish the depleted AT1 population. Age-related proteotoxic and oxidative stress, mitochondrial dysfunction, telomere shortening and epigenetic change render AT2 cells less able to undertake this repair, limiting regenerative capacity.^22^ Exposure of AT2 cells to SASP further impairs regeneration, and dysregulated ‘Wingless-related integration site’ (Wnt) signalling in the alveolar niche specifically affects AT2–AT1 cell transdifferentiation ability.^23^,^25^ Transcriptomic analysis of human lung has suggested that overall AT2 cell number decreases with age, but with disproportionate loss of the highest surfactant-producing ‘bulk’ AT2 cell subtype (AT2B) (vs the more stem-cell like AT2S population).^26^

#### Acquisition of mesenchymal features

Prolonged dysregulation of proteostasis, for example, due to expression of misfolding SFTPC variants, may lead to a phenotypic change of AT2 cells with acquisition of mesenchymal features.^27^ In health, this transient, self-limiting process allows changes in cell:cell junction and altered cell mobility to facilitate wound healing, but when permanent or in excess, it has profibrotic implications. Declining autophagy upregulates mesenchymal feature-promoting transcriptional programmes,^28^ as does a prolonged UPR^29^ which becomes more likely with age due to increasing misfolded protein burden, declining chaperone function and environmental influences. Components of SASP and the proinflammatory environment seen with ageing further promote the acquisition of mesenchymal features.^30^

#### Surfactant homeostasis

The amount and composition of pulmonary surfactant changes with age. The remaining AT2B cells in aged lungs produce and secrete less pulmonary surfactant than their younger counterparts,^20 26^ and this surfactant is relatively deficient in dipalmitoylphosphatidylcholine, the most abundant and key functional phospholipid. Oxidative stress may reduce the functionality of surfactant proteins,^31^ and declining efficiency of endocytic and degradative machinery further perturbs surfactant composition, reducing lung compliance (see ‘stretch’, below) and innate immunity afforded by surfactant proteins A and D (SFTPA/D).

#### Immune dysregulation

AT2 cells play an important role in the innate immune response, which becomes dysregulated with age. The global proinflammatory environment associated with ageing affects the ability of AT2 cells to sense pathogens via innate immune receptors such as toll-like receptors (TLRs), leading to an aberrant cytokine and chemokine response and an exaggerated or prolonged immune response.^32 33^ This is further exacerbated by the cumulative effects of oxidative stress on protein function and proteostasis. The effect of the ageing AT2 cell secretome on the alveolar niche also impacts on other immune cells including alveolar macrophages, which rely on exogenous cues from the alveolar lining fluid for their function.^34^ Downregulation of immunomodulatory SFTPA/D expression further drives the proinflammatory environment of the alveolar niche, as does cellular senescence and SASP production.

### Inherited genetic defects: fuelling the fire

In some individuals, inherited genetic variants render the alveolar epithelium extremely and prematurely vulnerable. Indeed, the identification and study of monogenic forms of pulmonary fibrosis (familial pulmonary fibrosis, FPF) has shaped the current hypothesis of IPF as triggered by the aberrant response of a vulnerable epithelium to injury.^35^ Implicated genes are divided into two distinct groups based on their function:

#### Telomere-related genes

These were first implicated in the pathogenesis of pulmonary fibrosis as part of the telomeropathy dyskeratosis congenita.^36^ It is now recognised that pathogenic variants in many telomere-related genes (TRGs), most commonly *TERT*, cause >50% cases of monogenic FPF, and genome-wide association studies (GWAS) have implicated common variants of TRGs in disease risk.^37^ Pathogenic TRG variants render the telomerase complex dysfunctional, leading to accelerated shortening of telomeres and exposing individuals to all the effects of premature epithelial ageing in the lung and other tissues.

#### Surfactant biology-related variants

A mutation in *SFTPC* was the first recognised monogenic cause of ILD.^38^ It is now appreciated that pathogenic variants in surfactant proteins and the machinery required for surfactant production cause FPF;^39^ most are dominantly inherited and confer a toxic gain-of-function in AT2 cells. The mechanisms involved largely mirror exaggerated forms of those seen with ageing, such as loss of proteostasis (through protein misfolding, abnormal trafficking or failure of degradation^29 40 41^) and altered surfactant composition; indeed, it was the study of these forms of FPF that has helped shape understanding of more generalised mechanisms.

In addition to TRGs, GWAS studies have implicated risk alleles associated with other candidate pathways including cell adhesion, host defence, mTOR signalling and cell cycle regulation; this provides further evidence of the broader mechanisms involved in disease.^42^

### Stretch: the effect of changing lung architecture with age

Ageing is associated with a decline in lung function even in healthy individuals. Changes in the underlying ECM result in reduced lung compliance and airspace enlargement, with alveoli becoming larger but less numerous.^43^ This causes increased alveolar epithelial stretch and is particularly problematic at the lung peripheries where there are relatively fewer AT2 cells per unit area and where physiological stretch during respiration is highest.^44^ Physiological stretch is crucial for normal alveolar epithelial function including surfactant secretion,^44^ but an excess of mechanical tension is deleterious. Stretched AT2 cells are more likely to acquire mesenchymal features,^45^ undergo cdc42-mediated cytoskeletal remodelling and failure of alveolar regeneration due to impaired AT2–AT1 cell differentiation.^46 47^ Together, these result in ongoing mechanical stress which upregulates profibrotic TGFβ_1_ secretion.^48^

## The pathological response of a vulnerable epithelium

The aged and/or genetically vulnerable epithelium is primed to react abnormally to exogenous challenges. Many of the fundamental perturbations associated with cellular ageing are exaggerated in IPF; fibrotic areas show high levels of ER stress even in the absence of expression of misfolding protein variants.^49 50^ Similarly, there is reduced autophagy,^51^ with accumulation of enlarged and dysmorphic mitochondria within IPF epithelium.^52^ Often thought of as ‘second hits’, environmental insults are usually inhaled and exacerbate aberrant behaviour of already vulnerable AT2 cells and ultimately lead to the development of IPF.^53^ Here, we discuss the consequences of epithelial injury caused by external insults that have been associated with the development of IPF, then dissect the dysregulated repair response which follows, leading to remodelling and fibrosis.

### Response to exogenous injury

#### Particulate matter

Epidemiological studies have established clear associations between the development of IPF and environmental and occupational exposures including cigarette smoke, metal/wood/silica/agricultural dusts and pesticides.^54^ A common feature of these substances is that they are a source of low size particulate matter (PM_2.5_), which is retained within lung parenchyma and can be entrained to epithelium.^55 56^ The high surface area of PM_2.5_ means toxic substances are highly adsorbed to the surfaces they make physical contact with, including cells within the alveolar niche.^57^ Here, they cause excess telomere shortening, epigenetic reprogramming, injury and chronic inflammation of epithelium consistent with an accelerated ageing phenotype.^58 59^ Further, environmental exposures may worsen mitochondrial dysfunction through the production of ROS and imbalanced proteostasis. The association of high PM_2.5_ exposure within the 30 days preceding IPF exacerbations highlights the role of these particles in both epithelial injury and vulnerability.^60^

#### Infectious insults

Exposure to infectious pathogens causes epithelial injury and may result in loss of epithelial barrier integrity and cytokine release. TLRs recognise pathogen and damage-associated molecular patterns (PAMPs and DAMPs) and are constituents of the first line of immune defence expressed on epithelial, stromal and immune cells.^61^ DAMPs and PAMPs are released as a result of epithelial injury and barrier disruption, and TLR sensing mediates the immune response. This sensing becomes less efficient with age,^33^ and interestingly, pathogenic variants encoding TLRs or adaptor proteins (eg, TOLLIP, TLR3 L412F) are associated with the development of IPF.^62^,^64^

In IPF, there are changes to the microbial communities colonising the airways, collectively termed the microbiome, which are associated with disease progression.^65^ A response to the presence of these bacterial communities is evident when studying the host peripheral blood gene expression profile.^66^ Epidemiological studies suggest a role of respiratory viral infections in triggering IPF,^67^ and animal work suggests their effects are more profound in the aged epithelium.^68^ Latent human herpes viruses (HHVs) inhibit TLR responses in infected human cells, thus compromising the normal innate immune response.^69^ HHVs can also trigger the UPR and thus may contribute to chronic vulnerability as well as acute injury.^70^ MtDNA released from injured, stressed cells can activate TLR9, and circulating mtDNA levels are associated with fibrosis progression.^71^

The exposures highlighted in this review are not exhaustive, but serve to highlight a common feature of IPF-associated exposures; they can contribute both to epithelial vulnerability as well as direct epithelial injury. Further, increasing vulnerability as injury progresses explains the observed precipitous decline and increased exacerbation rate seen in more advanced disease and in the presence of ongoing exposures.^72 73^

### Stalled and aberrant alveolar regeneration

In health, the regenerative response to lung epithelial injury involves a variety of progenitor populations including basal cells, club cells, submucosal gland duct cells, AT2 cells and bronchioalveolar stem cells.^74^ AT2 cells differentiate through transitional states via temporally and spatially co-ordinated molecular signalling to replace AT1 cells; this process is compromised in the ageing lung.^3^

In IPF, a phenomenon termed ‘bronchiolisation’ occurs, in which the alveolar epithelium is partially replaced by usually airway-specific epithelial cell types, especially keratin (KRT) 5-positive basal cells and ciliated cells.^75^,^77^ Animal studies have demonstrated that influenza-mediated injury to the alveolar epithelium triggers the proliferation and migration of distal airway (Krt5+/Trp63+) progenitor cells into damaged areas of lung parenchyma.^78 79^ The presence of alveolar-localised epithelial cells expressing progenitor cell markers (eg, KRT5, SCGB1A1) and transcriptional regulators of developmental genetic programmes (eg, YAP-TAZ, SOX9) is consistent with failed or dysregulated epithelial repair.^80 81^ Single-cell RNA sequencing (scRNA-seq) has provided greater granularity on this cellular heterogeneity,^77 82^ identifying two key cell types: a transitional epithelial population, characterised by KRT8 expression, and a KRT5−/KRT17+ population. Though not usually seen in health, perhaps due to their transient nature, the transitional population is able to terminally differentiate into AT1/AT2 alveolar epithelial cells.^83^ Conversely, the KRT5−/KRT17+ ‘aberrant basaloid cells’ express both epithelial and mesenchymal gene signatures^77 82 84^ and are not representative of a physiological intermediate. They are not identified in other age-related lung diseases such as chronic obstructive pulmonary disease, but do have a senescent and profibrotic phenotype, being a source of profibrotic cytokines (TGFβ_1_), ECM and SASP.^82 84^ KRT5−/KRT17+ basaloid cells can be induced in human precision cut lung slices treated with a profibrotic cocktail of cytokines.^85^ Age-dependent epithelial reprogramming has been demonstrated in a model of SARS-CoV-2 infection of human nasal epithelial cells, which resulted in an increase in KRT5+ basal cells and basaloid-like cells in older adult cultures compared with paediatric cultures.^86^

### Failure of wound healing and fibrosis propagation

Although the origin and pathological relevance of airway basal cell-like populations and aberrant basaloid cells in IPF remains unclear, it is hypothesised that their emergence represents an attempt to restore a functional mucosal barrier. There is cytokeratin upregulation in these cells, which alongside expression of ECM genes^82^ allows cell shape change and matrix secretion to re-establish a barrier. However, aberrant basaloid cells are not able to carry out homeostatic functions of normal alveolar epithelium, such as physiological surfactant expression and efficient gas exchange.

In addition to being a direct source of profibrotic cytokines (eg, connective tissue growth factor and TGFβ_1_), aberrant basaloid cells express high levels of integrin αvβ_6_. In a positive feed-forward loop, TGFβ_1_ signalling contributes to the development of the aberrant basaloid phenotype and increases αvβ_6_ expression^87^,^90^, which in turn increases the bioavailability of active TGFβ_1_ by releasing it from the latent TGFβ_1_ binding complex. This highlights how fibrosis may ‘spread’ and how disease progression may accelerate.^91^ Integrin αvβ_6_ is an attractive antifibrotic therapeutic target as its expression is minimal or absent in healthy epithelium, but increased in wound healing.^92^ Disappointingly, randomised control trials have not yet yielded positive results;^93^ this likely highlights the wider homeostatic importance of αvβ_6_-induced signalling in regulating inflammation and repair.

In summary, a highly irregular epithelial regenerative response to injury is observed in the fibrotic lung, characterised by the presence of dysregulated epithelial transitional cell states, cellular senescence and ECM accumulation (figure 1).

## Consequences of pathological behaviour: pathways to disease

**E**pithelial cells do not act alone in driving disease pathobiology in IPF, but in concert with the stromal and immune cells within their microenvironment.^94^ In this section, we introduce the powerful sequencing technologies that have been used to build ‘cell atlases’ of the fibrotic lung and provide valuable insight into changes in cell phenotype in the fibrotic niche. We then explore pathogenic crosstalk between aberrant alveolar epithelial cells and key cell types driving fibrosis. We refer to findings from several key mouse studies, acknowledging that there are differences in cellular composition and structure between the human and mouse lungs.^95^

### Dissecting the cellular microenvironment of the fibrotic lung with omics approaches

**Figure 2:**
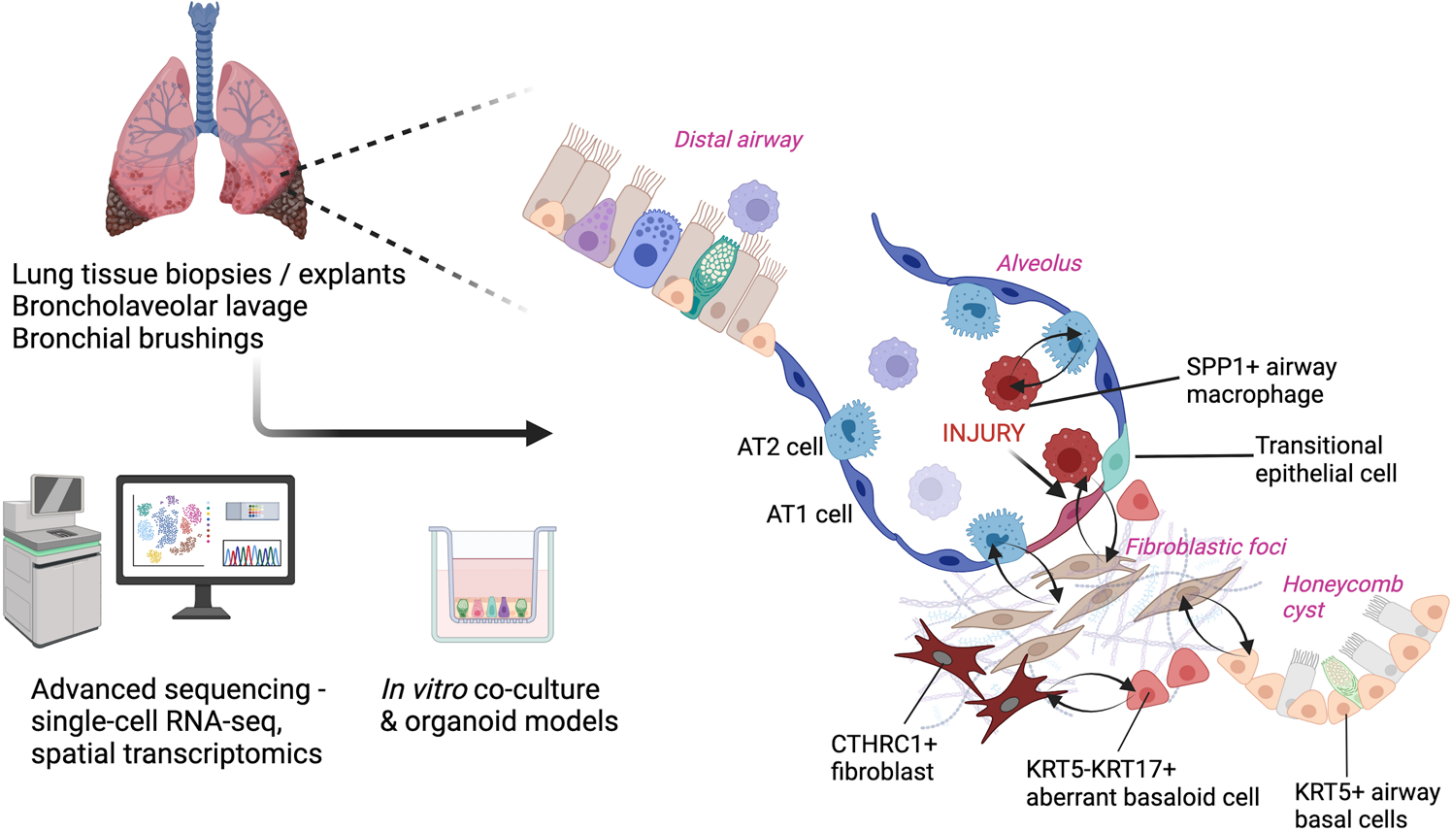
Decoding cell–cell interactions in the human fibrotic niche. Advanced sequencing tools and co-culture models can be used to study cell–cell interactions in pulmonary fibrosis. Schematic depicting examples of pathogenic cell types including SPP1+ airway macrophages, CTHRC1+ fibroblasts and KRT5-KRT17+ aberrant basaloid cells, with inferred cell–cell interactions driving pulmonary fibrosis. Created in BioRender. AT1, alveolar type 1 epithelial cell; AT2, alveolar type 2 epithelial cell; CTHRC1+, collagen triple helix repeat containing 1+; KRT5, keratin 5; SPP1, secreted phosphoprotein 1 (osteopontin).

In the lung ageing cohort study, ‘bulk’ RNA-sequencing of pooled cells from donor human lung tissue at different ages across the adult lifespan demonstrated upregulation of cell senescence markers and molecular pathways associated with mesenchymal activation and fibrosis in aged lung.^96^ Cell type deconvolution revealed a decrease in the proportion of epithelial cells and an increase in fibroblasts with lung ageing.^96^ Our knowledge of cell type diversity, cell states and cell–cell interactions within the fibrotic lung has significantly advanced owing to a growing number of scRNA-seq studies as previously discussed^77 82 97^ (figure 2). There are some disadvantages to this approach, however, including the introduction of bias due to loss of certain cell types during their dissociation from intact tissue, inconsistent cell type annotations between studies and loss of spatial location information for each cell, making it more difficult to contextualise the data in the complex microenvironment of the fibrotic lung.^98^ Spatial transcriptomics overcomes this last issue by enabling quantification of spatially-resolved gene expression mapped to specific locations within intact lung tissue sections, providing the positional context of cells in lung tissue.^99 100^ Using computational approaches, cell–cell communication networks in the lung microenvironment can be inferred from scRNA-seq data sets.^100 101^ When interpreting the data from these studies, it is important to consider the following limitations: (1) sample numbers are often limited by prohibitive costs or difficulty procuring tissue, (2) the lung tissue used is often derived from end-stage IPF and therefore does not provide insight into early disease pathogenesis and (3) cell–cell interactions are based on computational predictions, so are inferred rather than definitive and require validation at the protein level or through functional experiments in vitro. Human two-dimensional and three-dimensional in vitro co-culture models have been employed as powerful tools to further dissect cell–cell interactions.^102^

### Epithelial–fibroblast crosstalk

Bidirectional crosstalk between alveolar epithelial cells and fibroblasts is essential to the lung injury repair response, and when dysregulated, can drive fibrogenesis.^103^ Studies in mice show signals from adjacent fibroblasts termed ‘mesenchymal alveolar niche cells’, expressing *Axin2* and *Pdgfrα,* support the self-renewal and differentiation of AT2 cells to AT1 cells.^104^ Furthermore, ablation of *Scube2-*expressing alveolar fibroblasts, which make physical contact with AT2 cells, results in AT2 cell loss.^105^ Using scRNA-seq, four distinct fibroblast subtypes were discovered, each residing in different anatomical regions of the IPF lung including the airway subepithelial and alveolar regions.^77^ An ‘interactome’ analysis suggested epithelial cells signal to fibroblasts using a complex network of growth factors, chemokines and cytokines through integrin receptors.^77^

Analysis of collagen-producing mesenchymal cells in the lung using scRNA-seq has revealed a pathologic ‘collagen triple helix repeat containing 1’ (*CTHRC1)+* fibroblast population in patients with IPF.^106^ These arise from alveolar fibroblasts, localise to the leading fibrotic edge in fibroblastic foci and express high levels of ECM-related genes.^106^ Importantly, pathologic *CTHRC1-*high fibroblasts also emerge when adult human lung mesenchymal cells are co-cultured with human AT2 cells.^89^ Profibrotic *CTHRC1-*high fibroblasts are co-localised with airway basal cell-like populations and aberrant basaloid cells in the IPF lung.^89 100^ In an animal model, ablation of these fibroblasts reduces collagen content and lung fibrosis severity.^105^

IPF airway basal cells, which are expanded as a feature of ‘bronchiolisation’ in the fibrotic lung, exhibit transcriptional reprogramming with increased stem cell markers and form tube-like structures resembling bronchospheres in a three-dimensional organoid culture model.^84^ IPF airway basal cells stimulate increased human lung fibroblast (HLF) proliferation and collagen production in vitro.^84^ Basal cells have been found to secrete WNT7A, which activates HLFs to produce fibronectin and in turn inhibits AT2 progenitor cell renewal in vitro.^107^ There is also reciprocal fibroblast-to-epithelial cell crosstalk where TGF-β_1_ produced by HLFs upregulates sFRP2, a soluble modulator of WNT signalling, which induces AT2 cells to transdifferentiate to KRT5+ basal like cells in an organoid model.^108^ IPF HLFs were also shown, via interleukin (IL)-6 signalling, to induce airway epithelial barrier dysfunction, a change in cell type proportions and persistent ‘fluidisation’, where epithelial cells collectively adopt a more migratory programme.^109^

The ECM microenvironment also plays a critical role in influencing epithelial cell activity in the fibrotic lung. ECM derived from IPF HLFs restricts migration of KRT5+ basal cells in vitro and induces upregulation of genes associated with fibrosis.^110^ The ECM matricellular protein ‘Secreted Protein Acidic and Rich in Cysteine’ (SPARC) is localised to IPF fibroblastic foci,^111^ is overexpressed in IPF HLF-derived ECM,^110^ disrupts epithelial barrier integrity^112^ and modulates migration of AT2 cells^112^ and KRT5+ basal cells in vitro.^110^

Together, these studies illuminate interactions between airway and alveolar epithelial cell populations, HLFs and the ECM microenvironment which can determine cell phenotype, function and pathways to pulmonary fibrosis and lung remodelling.

### Epithelial–immune cell crosstalk

Immune cells play a central role in the pathogenesis of IPF,^113^ and our knowledge of immune cell subsets residing in the fibrotic lung has been enriched through scRNA-seq atlases.^82 114^ Airway macrophages are the most abundant immune cell in the lung and are positioned at the interface of epithelium and environment,^115^ so will be focused on here. Alveolar macrophages phagocytose inhaled particulates and apoptotic cells, promote immune tolerance to innocuous inhaled stimuli and provide a robust immune response to pathogens.^115^ A single-cell atlas of mouse lung ageing revealed upregulation of genes linked to lung injury and fibrosis, including *Spp1* in alveolar macrophages of old mice.^116^ Human scRNA-seq studies have identified airway macrophage heterogeneity in the IPF lung^82 114 117^ and exposed a profibrotic airway macrophage subset expressing *SPP1*, which localises to fibrotic regions.^117^ Integration of multiple lung disease data sets in the Human Lung Cell Atlas revealed *SPP1+* profibrotic monocyte-derived macrophages in the IPF samples but also in later-stage COVID-19 and lung cancer, suggesting this profibrotic macrophage subset is shared across disease states.^118^ Macrophage phenotype depends heavily on signals from the microenvironmental niche,^119^ but the extent to which the lung epithelium is able to modulate polarisation of macrophage phenotype remains poorly understood.

Spatial transcriptomics has revealed IPF-tissue specific cellular niches including an ‘airway macrophage niche’ within the airway lumen consisting of *SPP1+* macrophage subsets colocalised with epithelial cell populations including aberrant basaloid cells, preterminal bronchial secretory cells and distal *SFTPB+-*ciliated cells.^100^ Cell–cell communication analysis identified specific ligand–receptor interactions between airway niche cell types, such as expression of *SCGB3A2* by epithelial cell subtypes, binding to ‘macrophage receptor with collagenous structure’ (MARCO) expressed by macrophages.^100^ This study highlights novel epithelial–macrophage interactions in distinct cellular niches of the IPF lung.

Interactions between epithelial cells, macrophages and other immune cells in the ageing human lung and IPF remain poorly understood. It has been shown that AT2 cell injury in a mouse model resulted in ‘C-C Motif Chemokine Receptor 2’ (CCR2)-mediated accumulation of non-resident CD11b+ macrophages and ‘lymphocyte antigen 6 family member C’ (Ly6C)^hi^ monocytes, driving lung fibrosis.^120^ Treatment of mouse AT2 cells with TGF-β_1_ in vitro induces AT2 cell senescence, with secretion of mediators including cytokines IL-4 and IL-13 which upregulate profibrotic gene expression changes in alveolar macrophages.^121^ Pretreatment of mouse AT2 cells with bleomycin induces expression of Sonic hedgehog, a key developmental signalling protein, promoting polarisation of alveolar macrophage to a profibrotic phenotype via SPP1.^122^ Macrophages have also been reported to interact directly with myofibroblasts in IPF. Adherens junction protein cadherin-11 (CDH11) facilitates interactions between activated CD68-positive macrophages and myofibroblasts within fibroblastic foci.^123^ In mice, contact achieved by CDH11-mediated adhesion allows latent TGF-β_1_ produced by macrophages to be activated by lung myofibroblasts, maintaining a profibrotic niche.^123^

### Concluding remarks

Processes linked to ageing cause epithelial dysfunction and vulnerability which critically primes the lung to a fibrotic response to injury. Alveolar and airway basal epithelial cell subsets communicate with an array of mesenchymal and immune cell subsets that reside in the fibrotic niche, to drive disease pathobiology. Further research focused on unravelling epithelial cell types and states in preclinical/early disease at the onset of fibrosis will be pivotal in shaping future, preventative therapies for this devastating age-related disease.

### Search strategy

We used PubMed to search the MEDLINE database for articles published over approximately the last 10 years, with the terms “idiopathic pulmonary fibrosis ”, “lung fibrosis”, “ageing”, “cellular senescence”, “alveolar epithelial cells”, “fibroblasts”, “single-cell transcriptomics”, “spatial transcriptomics”, “cell-cell communication” to identify pertinent articles. We included only publications published in English, focused primarily on work involving human samples and in vitro models of disease, and selected those with findings that were, in our view, of greatest importance in view of the limitation on the number of references. We also included highly regarded older publications where applicable.
